# Identification of a Rare EGFR T790I Mutation in Lung Adenocarcinoma Sensitive to Osimertinib

**DOI:** 10.3389/fonc.2021.727312

**Published:** 2021-10-20

**Authors:** Yu Wang, Songtao Liu, Alei Feng, Huan Luo, Jinwei Hu, Kai Wang, Wei Dong

**Affiliations:** ^1^ Department of Oncology, Shandong Provincial Hospital Affiliated to Shandong First Medical University, Jinan, China; ^2^ OrigiMed Inc., Shanghai, China; ^3^ Department of Thoracic Surgery, Shandong Provincial Hospital, Cheeloo College of Medicine, Shandong University, Jinan, China; ^4^ Department of Thoracic Surgery, Shandong Provincial Hospital Affiliated to Shandong First Medical University, Jinan, China

**Keywords:** lung adenocarcinoma (AC), T790I, osimertinib, EGFR, target therapy

## Abstract

Estimated glomerular filtration rate (EGFR)-sensitive mutations are extremely important for targeted treatment strategies in lung cancer. Osimertinib can effectively inhibit the activity of EGFR-sensitive mutations, including the T790M mutation. However, the efficiency of osimertinib for rare mutation types of T790 is unclear. Here, we report the case of a Chinese patient with lung adenocarcinoma (LADC) harboring a T790I mutation who achieved significant benefits from osimertinib treatment.

## Introduction

**Figure 1:**
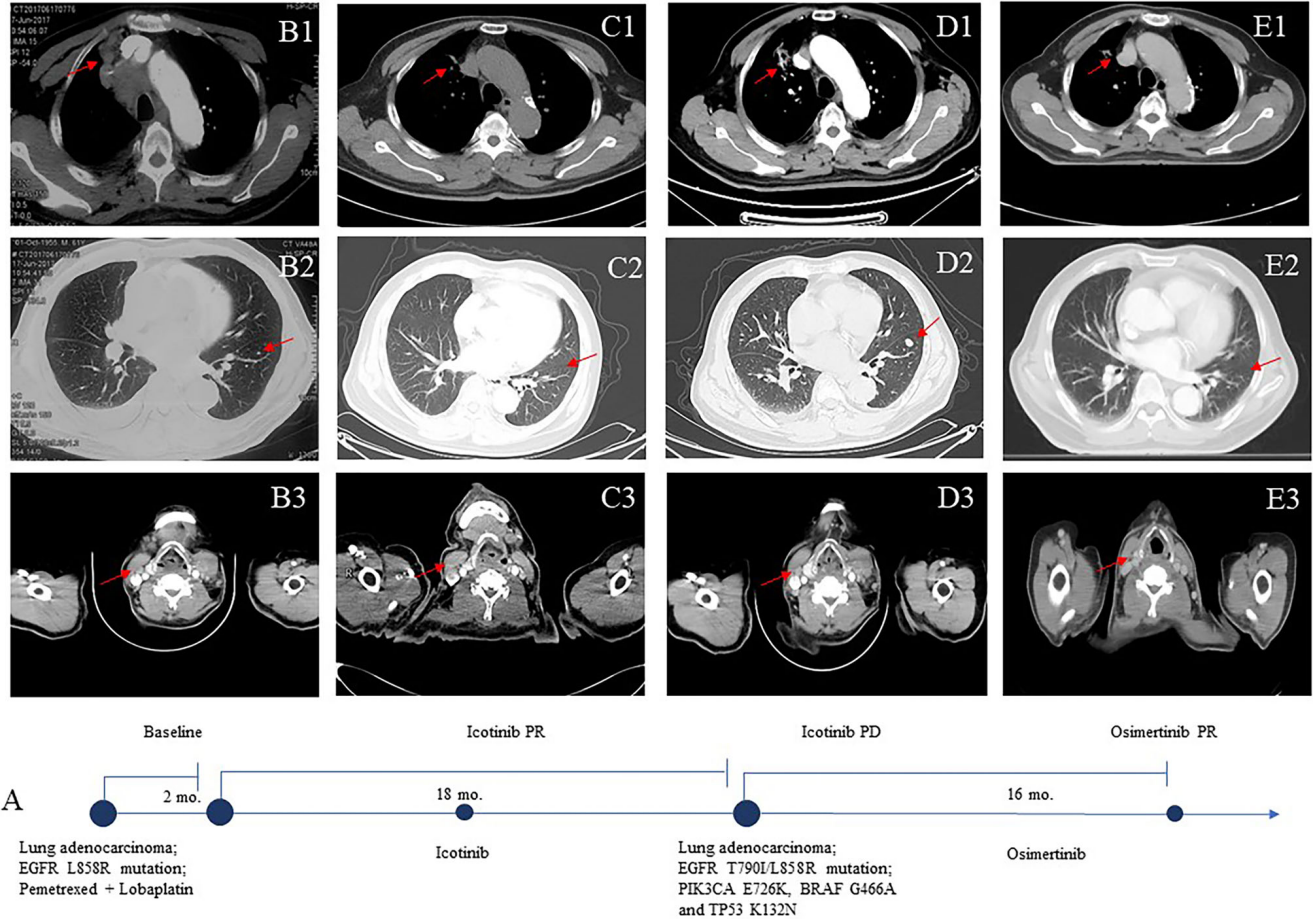
Computed tomographic (CT) images **(B**–**E)** of the patient during treatment course **(A)**. Baseline images at diagnosis (June 2017) **(B1**–**3)**. Images after icotinib treatment **(C1**–**3)**. The patient’s upper lobe of the right lung, mediastinal and right hilar lymph nodes, bilateral pulmonary nodules, and right supraclavicular region were smaller. Eighteen months after icotinib treatment disease progression **(D1**–**3)**. The patient achieved a sustainable clinical benefit from osimertinib treatment **(E1**–**3)**.

Estimated glomerular filtration rate (EGFR)-tyrosine kinase inhibitors (TKIs) are a standard treatment option for patients harboring activating EGFR mutations, and several TKIs that specifically target EGFR have been approved by the FDA (1). However, many patients develop acquired drug resistance because of the EGFR T790M mutation (2). Although osimertinib can effectively inhibit the activity of the EGFR-sensitive mutation T790M (3), the efficiency of osimertinib for the rare mutation type T790 is unclear; therefore, the recognition and clinical evidence of rare mutations is essential for precision oncology. Hence, we report a Chinese patient with lung adenocarcinoma (LADC) with a novel EGFR T790I mutation, which is a rare predicted first-generation TKI-resistance mutation. He achieved significant benefits accompanied by undetectable T790I expression after osimertinib treatment (**Figure 1A**).

## Patient and Sequencing

This study was approved by the institutional review board and conducted following the tenets of the Declaration of Helsinki. Informed consent was obtained from the patient. Formalin-fixed paraffin-embedded (FFPE) tumors and matched blood samples from a 61-year-old Chinese male with LADC were collected and transferred to OrigiMed (Shanghai, China) for genetic alteration detection.

Genomic DNA of tumor samples and white blood cells from matched blood was extracted from the FFPE tumor and blood samples using the QIAamp DNA FFPE Tissue Kit and QIAamp DNA Blood Midi Kit (Qiagen, Hilden, Germany) according to the manufacturer’s instructions. The testing was carried out in a laboratory certified by the College of American Pathologists and Clinical Laboratory Improvement Amendments.

## Results

**Figure 2:**
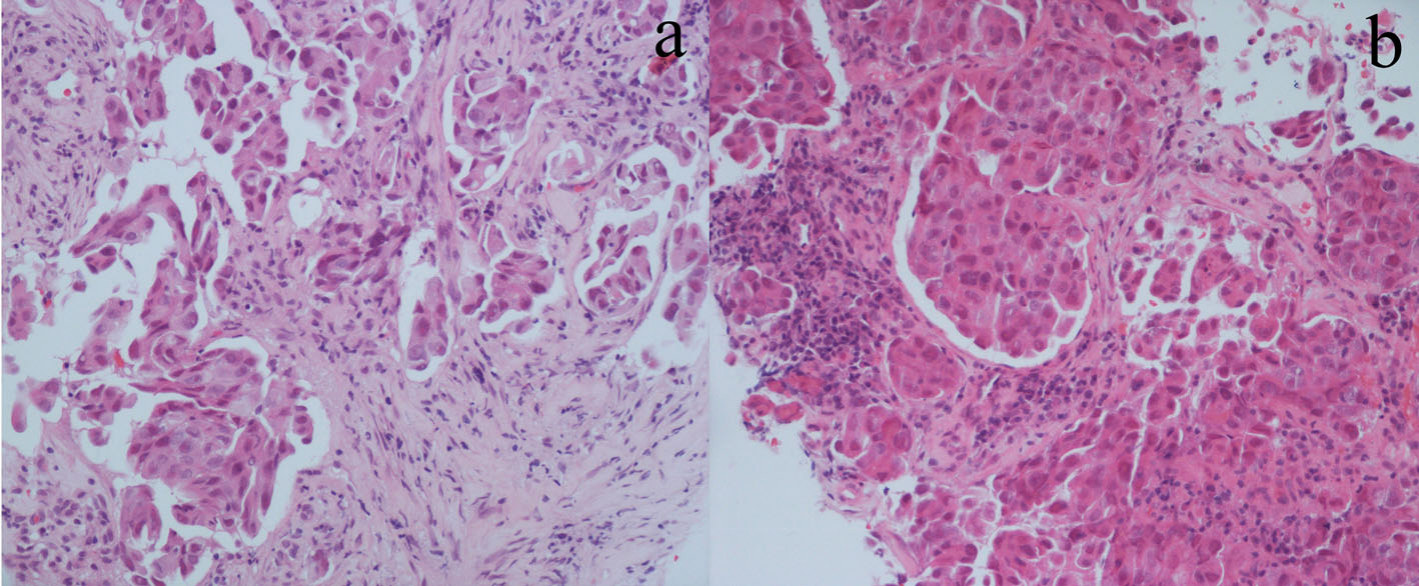
Hematoxylin and eosin staining of the first **(A)** and repeated **(B)** biopsy specimens.

The patient was a 61-year-old Chinese man without a history of smoking or family history of lung cancer. He had been receiving systemic treatment for coronary heart disease for 13 years. This patient was admitted with a 2-month history of chest tightness and asthmatic and irregular coughing in June 2017. Chest computed tomography (CT) revealed a space-occupying lesion in the upper lobe of the right lung, which invaded the mediastinal and right hilar lymph nodes and right supraclavicular region (**Figure 1B**). Right supraclavicular lymph node biopsy was performed, and pathological examination allowed diagnosis of lung adenocarcinoma T2N3M1a (**Figure 2A**), and immunohistochemistry (IHC) revealed the tumor to be NapsinA+, TTF-1+, CK7+, CD10−, and vimentin+. The EGFR L858R mutation was detected using a conventional amplification-refractory mutation system. The patient initially received intravenous pemetrexed combined with lobaplatin but could not tolerate after one cycle; thus, he was administered icotinib (125 mg three times daily) (**Figure 1C**) in August 2017.

**Figure 3:**
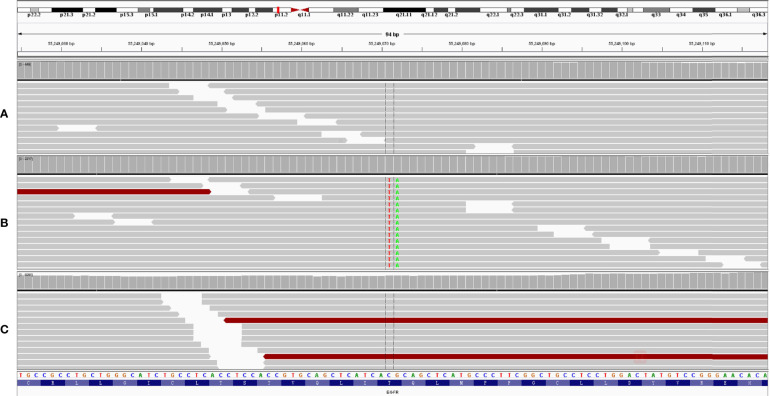
A novel EGFR T790I mutation was detected after icotinib treatment for 18 months **(B)**. This mutation disappeared after osimertinib treatment for 16 months **(C)**. White blood cell was used as control **(A)**.

In February 2019, after treatment with icotinib for 18 months, a CT scan showed disease progression (**Figure 1D**) with multiple bilateral pulmonary nodules and multiple mediastinal and lymph node metastases. A lung biopsy after radiographic disease progression showed adenocarcinoma again (**Figure 2B**). After obtaining consent from the patient, his FFPE tumor sample was sent to the OrigiMed (Shanghai, China) for next-generation sequencing analysis with a YuanSu™ panel (4). The T790I (c.2369_2370delinsTA) mutation, a rare variant which causes a threonine (T) to isoleucine (I) missense mutation at codon 790, was identified (**Figure 3**). Other mutations were also detected, including EGFR L858R, PIK3CA E726K, BRAF G466A, and TP53 K132N. The patient was started on osimertinib (80 mg once daily) treatment in March 2019 with good tolerance and treatment for 16 months. After a follow-up in July 2020, a partial response (RECIST 1.1) was obtained for osimertinib treatment, and the mediastinal and lymph nodes disappeared (**Figure 1E**). The next-generation sequencing results of cell-free DNA showed that both the EGFR T790I and L858R mutations were negative. Up to the date of this report, the patient is still receiving osimitinib treatment, and his condition is stable without significant progress.

## Discussion

The “gatekeeper” residue threonine 790 is an important determinant of inhibitor specificity in the ATP-binding pocket as increased ATP affinity is the primary mechanism by which the T790M mutation confers drug resistance (5, 6). T790I may also act as a “gatekeeper” mutation to activate kinase activity in receptor tyrosine kinases. A computational exploration of “gatekeeper” mutations in EGFR revealed that EGFR T790M and EGFR T790I stabilize both the active and inactive forms of the kinases (7). The oncogenic activity of the T790I mutation has previously been reported in Ba/F3 cells (8) but has not been observed in TKI-refractory lung cancers in clinics. A previous study revealed that osimertinib can inhibit the growth of transgenic lung cancer mouse models harboring mouse EGFR exon19 deletion (mDEL) tumors with T790I mutation; however, these tumors acquired resistance to osimertinib and regrew 2 months after treatment (9). In our case, after disease progression was caused by the development of drug resistance to a first-generation EGFR TKI, the patient achieved partial response and progression-free survival after osimertinib treatment. Although osimertinib can effectively inhibit the activity of EGFR-sensitive mutations and T790M (10), a number of other tertiary EGFR mutations have been observed to confer osimertinib resistance. Similar to EGFR T790M, the T790I mutation confers resistance to icotinib; however, in this case, T790I appears to have a weaker resistance mutation than T790M and loses activation of both EGFR and downstream signaling following osimertinib treatment.

This is the first report of an LADC patient with EGFR T790I mutation who achieved a good response with osimertinib, thereby presenting the first clinical evidence of the efficacy of osimertinib, targeting T790I, which indicated that T790I is a candidate osimertinib-sensitive mutation. The result is an individualized treatment, maybe a limitation of this study, but also demonstrates the precision therapies being crucial for optimal individualized management and thus elevating the survival rate. Furthermore, our findings highlight the importance of dynamic genetic monitoring to tailor precision therapies to improve clinical outcomes.

## Data Availability Statement

The datasets presented in this study can be found in online repositories. The names of the repository/repositories and accession number(s) can be found in the article/supplementary material.

## Ethics Statement

The studies involving human participants were reviewed and approved by the ethics committee of Shandong Provincial Hospital. The patients/participants provided their written informed consent to participate in this study. Written informed consent was obtained from the individual(s) for the publication of any potentially identifiable images or data included in this article.

## Author Contributions

WD and YW had full access to all of the data in the manuscript and take responsibility for the integrity of the data and the accuracy of the data analysis. Concept and design: all authors. Acquisition, analysis, and interpretation of data: all authors. Manuscript drafting: WD and YW. Critical revision of the manuscript for important intellectual content: all authors. Statistical analysis: YW and AF. Supervision: WD. All authors contributed to the article and approved the submitted version.

## Funding

This work was supported by the National Natural Science Foundation of China (81802282) and the Jinan Science and Technology Development Program (201907112).

## Conflict of Interest

Author HL, JH and KW were employed by the OrigiMed Inc.

The remaining authors declare that the research was conducted in the absence of any commercial or financial relationships that could be construed as a potential conflict of interest.

## Publisher’s Note

All claims expressed in this article are solely those of the authors and do not necessarily represent those of their affiliated organizations, or those of the publisher, the editors and the reviewers. Any product that may be evaluated in this article, or claim that may be made by its manufacturer, is not guaranteed or endorsed by the publisher.
